# Supplementary material for: Development and validation of a depression risk prediction nomogram for US Adults with hypertension, based on NHANES 2007–2018
Source: PLoS One. 2023 Apr 5;18(4):e0284113. doi: 10.1371/journal.pone.0284113 (PMC10075444; doi:10.1371/journal.pone.0284113)
Supplement: Attachment — Submitted filename: Response to Reviewers.docx [file pone.0284113.s002.docx]

Dear Dr. Gregor Stiglic and dear reviewers,

Re: Manuscript ID: PONE-D-22-34347 and Title: Development and validation of a depression risk prediction nomogram for US Adults with hypertension, based on NHANES 2007-2018

Thank you for your letter and the reviewers’ comments concerning our manuscript entitled “Development and validation of a depression risk prediction nomogram for US Adults with hypertension, based on NHANES 2007-2018”. Those comments are valuable and very helpful. We have read through comments carefully and have made corrections. Based on the instructions provided in your letter, we uploaded the file of the revised manuscript. We have marked in red the parts that need to be revised as mentioned by the reviewers. The remaining parts that we have revised are marked in blue. A point-by-point response to the two nice reviewers is listed below this letter. The reviewers' comments are listed below in italics and the specific questions are numbered. Our responses are given in normal type.

We would love to thank you for allowing us to resubmit a revised copy of the manuscript and we highly appreciate your time and consideration.

Sincerely.

Yan, Zhang

15, Mar, 2023

**Replies to the reviewers’ comments:**

**Answers to REVIEWER #1:**

**Q1**. *In the methods section there are listed all the patients that have been excluded form study population. In my opinion it is a simple list and it is not clear on which criteria some patients has been excluded. If some literature references are available, please cite them in the text*.

Response：Thank you very much for double checking, this was an oversight on our part, we have listed the inclusion and exclusion criteria for the study population on page 7, lines 10 and 11, and we have also added a flowchart for the screening of study participants (see Fig1). Thank you for the heads up.

**Q2**. *In the discussion section it is explained how the nomogram could be used to obtain the probability of risk of developing depression in patients with hypertension. However, depression is a multifactorial disease. While nomogram could give a probability risk of developing depression, it could be interesting to briefly explore (maybe revising literature) how it could be implemented in the everyday clinical practice*.

Response：Thank you for your suggestion, we have added a discussion of how nomogram is implemented in clinical practice on page 29, lines 1 to 9, and cited the "Guide to presenting clinical prediction models for use in clinical settings clinical settings, BMJ (Clinical research ed.) 365 (2019) l737" to explain how the clinical prediction models of nomogram can be applied in clinical practice.

**Q3**. *Finally, at the end of the discussion section it is mentioned how the study could be affected by a selection bias regarding the study population, please better specify what the authors mean.
I would suggest a reworking of the discussion sections of this paper & then re-submit*.

Response：Thank you for underlining this deficiency. NHANES is a large database with many indicators, and based on our current limited understanding, we were unable to include all covariates related to hypertension and depression; Therefore, some variables may have been omitted in our selection of included variables and thus would have been subject to selection bias in the selection of variables. We have also included an explanation of selection bias on page 29, lines 13, and page 30, lines 1 to 4.

**Answers to REVIEWER #2:**

**Q1**. *Why females are more depressed than males? This need to be discussed in the manuscript*.

Response：Thank you for your suggestion. We have further refined the discussion on the relationship between gender and depression on page 25, lines 9 to 13 and page 26, line 1 to 2, and we have also cited "Sex differences in anxiety and depression: role of testosterone, Frontiers in neuroendocrinology 35(1) (2014) 42-57" to explain the mechanism, which mentions that the gender difference in depression between women and men is caused by the different amount of testosterone produced by men and women.

**Q2**. *The mean age of subjects is 54.06 years. Therefore, is it associated with menopause?*

Thanks for your great suggestion on improving the accessibility of our manuscript. In our study, depression was mostly concentrated in middle-aged and older adults, and we also found some literature mentioning that older patients are more likely to be depressed. Moreover, the literature “The association of widowhood and living alone with depression among older adults in India, Scientific reports 11(1) (2021) 21641” and “Loneliness and Depression Among Older Adults in Urban Subsidized Housing, Journal of aging and health 30(3) (2018) 458-474” also mention that as people age, the cumulative effects of several adverse factors may increase the risk of negative mental health outcomes, which can eventually cause depression. Moreover, previous studies have concluded that women are more likely to be depressed than men, so women may be more likely to suffer from depression during menopause. We have added discussion on page 25, lines 9 through 13, and page 26, lines 1 through 11, combining the effects of age and gender on depression.

**Q3**. *The mean weekly sleep is 7.11 hours for depressed individuals. If this number is correct, are they also associated with some other sleep related disorder?*

Response：We apologize for our carelessness, it was a spelling mistake on my part, I spelled "sleep time on workdays" as "weekly sleep", After a thorough review of the full text, we have corrected all "weekly sleep" to "sleep time on workdays" in the resubmitted manuscript. Thank you for the correction.

**Q4**. *Discussion part is not very well written. It has to be elaborated and discuss every finding made by the authors.*

Response：We sincerely thank you for your valuable comments. In the discussion section, we present a more thorough and extensive description of all the nomogram predictors based on our findings. We've discussed and found pertinent literature to discuss for each of the study's findings.
